# Elevated circulating IL-8 correlates with poor prognosis in urological cancers: a meta-analysis and bioinformatic validation

**DOI:** 10.1080/07853890.2025.2486592

**Published:** 2025-04-03

**Authors:** Yuxuan Lin, Yonghe Liao, Mengfan Huang, Jinhai Shen

**Affiliations:** ^a^ Department of Pharmacy, Guangxi Hospital Division of The First Affiliated Hospital, Sun Yat-sen University, Nanning, PR China; ^b^ College of Pharmaceutical Science, Guangxi Medical University, Nanning, PR China; ^c^ State Key Laboratory of Natural Medicines, China Pharmaceutical University, Nanjing, PR China; ^d^ Center for New Drug Safety Evaluation and Research, China Pharmaceutical University, Nanjing, PR China

**Keywords:** Interleukin-8, urological cancers, biomarker, prognosis, meta-analysis

## Abstract

**Background:**

Interleukin-8 (IL-8) is a key cytokine that has been implicated in multiple aspects of cancer progression and therapeutic resistance. Elevated levels of circulating IL-8 (cIL-8) have been implicated in adverse clinical outcomes among patients with urological cancers. However, definitive evidence consolidating these observations remains lacking. The present study aims to synthesize the existing research findings to provide a comprehensive, evidence-based reference for clinical practice.

**Methods:**

A systematic literature search was conducted to identify relevant studies that reported on the prognostic impact of cIL-8 levels in urological cancer patients. Hazard ratios (HRs) for overall survival (OS) and progression-free survival (PFS) were extracted and pooled to estimate the overall effect. Furthermore, Kaplan–Meier’s survival analyses were conducted using RNA-seq data from The Cancer Genome Atlas (TCGA) through the Gene Expression Profiling Interactive Analysis 2 (GEPIA 2) online tool to validate the observed associations.

**Results:**

A total of 19 cohorts encompassing 2740 patients from 12 studies were included in the meta-analysis. The findings revealed that elevated cIL-8 levels were significantly associated with inferior OS (HR: 1.86; 95% confidence intervals (CI): 1.72–2.02) and PFS (HR: 1.59; 95%CI: 1.25–2.03) in patients with urological cancers. The consistency and validity of these results were further supported by survival analyses performed using the GEPIA 2 tool.

**Conclusions:**

This study, which is the first meta-analysis to systematically examine the prognostic significance of cIL-8 in urological cancers, supported by bioinformatics validation, confirms that elevated cIL-8 levels serve as a potential biomarker for predicting adverse outcomes. Our findings underscore the importance of targeting IL-8 as a therapeutic strategy to overcome treatment resistance and improve outcomes for urological cancer patients. Further research into IL-8-targeted therapies and their integration into clinical practice is urgently needed to enhance the treatment landscape for urological cancers.

## Introduction

Urological cancers, encompassing a spectrum of malignancies affecting the genitourinary system, pose a formidable health challenge globally [1]. The mortality rates associated with these cancers, including urothelial carcinoma (UC) and renal cell carcinoma (RCC), underscore the urgency for precision medicine approaches to improve patient outcomes. The advent of targeted therapies and immunotherapies has heralded a new era in urological cancer treatment, offering hope for patients with advanced disease [2,3]. However, the emergence of treatment resistance remains a significant hurdle in achieving optimal therapeutic responses for all patients [4,5]. Therefore, a deeper understanding of the mechanisms driving treatment resistance is crucial for the identification of predictive biomarkers, which can inform personalized treatment approaches and ultimately enhance patient outcomes.

Interleukin-8 (IL-8), a chemokine of significant importance in the pathogenesis of various cancers, is primarily secreted by tumour cells, endothelial cells and immune cells [6,7]. IL-8 has significant potential as a biomarker for early cancer detection [8,9]. Elevated levels of IL-8 in the circulation may indicate the presence of cancer at an early stage, allowing for earlier intervention and potentially better outcomes. Emerging evidence indicates that IL-8 is a pivotal factor in the development of resistance to both targeted and immunotherapeutic interventions [10,11]. Mechanistically, elevated concentrations of IL-8 are implicated in the recruitment of immunosuppressive cell types, including myeloid-derived suppressor cells (MDSCs), tumour-associated macrophages (TAMs) and neutrophils, which can subvert the adaptive T-cell response [6,10,12]. Furthermore, IL-8 has the capacity to directly stimulate cancer cell proliferation and to foster angiogenesis, thereby constructing a tumour microenvironment that is inhospitable to effective immunotherapy [6,10]. Additionally, IL-8 interacts with other key cytokines and signalling pathways, such as the CXCL family and vascular endothelial growth factor pathway, amplifying its impact on tumour vascularization and immune modulation [6,10]. Therefore, targeting IL-8 or its signalling pathway may be a promising strategy to overcome treatment resistance and improve outcomes for cancer patients [13,14]. Several existing therapies aim to target IL-8 or related pathways. For example, both anti-IL-8 antibodies and inhibitors of the CXCR1/2 receptors have been developed and investigated as potential cancer treatments [13,14]. However, these therapies face certain limitations. Incomplete efficacy, the development of resistance over time, and challenges in translating these therapies from preclinical studies to clinical practice are some of the issues that need to be addressed. Understanding these limitations is crucial for the development of more effective targeted interventions.

Although numerous studies have indicated a correlation between elevated levels of circulating IL-8 (cIL-8) and poor prognostic outcomes in urological cancers, these studies often have small sample sizes, and their results are inconsistent. Given these limitations and the clinical importance of targeting cIL-8 in urological cancer treatment, a systematic review and synthesis of the available literature are urgently needed to clarify the prognostic value of cIL-8 in urological cancers. In this study, we aim to consolidate existing clinical evidence through a meta-analysis with bioinformatic analysis, thereby offering a more robust, evidence-based foundation for guiding personalized care and treatment strategies for patients with urological cancers.

## Methods

### Protocol and guideline

This meta-analysis has been prospectively registered in PROSPERO under the identifier CRD42024585894 and was conducted in strict compliance with the PRISMA 2020 reporting criteria [15].

### Data sources and search strategy

The databases PubMed, Scopus and Embase were comprehensively searched from their inception through 25 August 2024, to identify all relevant studies. The search strategy employed a combination of MeSH terms and free-text keywords, including ‘interleukin-8,’ ‘urothelial carcinoma’ and ‘renal cell carcinoma.’ A detailed list of these search terms is provided in Table S1.

### Inclusion criteria

All available research, including published articles, conference abstracts and poster presentations, that investigated prognosis in patients with urological cancers by comparing those with high levels of cIL-8 to those with low levels, was initially screened for inclusion. Studies were eligible for the meta-analysis if they reported hazard ratios (HRs) with corresponding 95% confidence intervals (CIs) for either overall survival (OS) or progression-free survival (PFS). The selection process involved an initial examination of titles and abstracts by two independent reviewers, followed by a thorough review of the full-text articles to establish their eligibility. In cases where inconsistencies or disagreements emerged during the eligibility assessment, a third researcher was involved in the re-evaluation of the contentious study to make the final decision regarding its inclusion.

### Data extraction and quality assessment

The impact of cIL-8 levels on prognosis was evaluated in terms of OS and PFS for patients with urological cancers. Data extraction and summary were performed by a single investigator and subsequently verified by an independent reviewer. In instances where both univariate and multivariate analyses were presented, preference was given to the multivariate data for inclusion. The information extracted included details such as study design, the number of patients with available cIL-8 data, cIL-8 cutoff value and detection method, treatment regimen, cancer type and stage, as well as HRs with their associated 95%CIs for OS and PFS. The quality of the studies included in the analysis was independently assessed by two authors using the modified Newcastle-Ottawa Scale (NOS) [16].

### Statistical analysis

Pooled estimates for OS and PFS were derived by synthesizing HRs along with their corresponding 95%CIs. The *I*^2^ statistic and the Cochran’s *Q* test were employed to assess the degree of heterogeneity among the studies. In instances where the *I*^2^ value exceeded 50% and the *p* value was less than .1, signifying considerable heterogeneity, a random-effects model was adopted. Conversely, a fixed-effects model was applied when heterogeneity was not significant. In cases where discrepancies emerged between the results of the two models, the random-effects model was favoured due to its greater conservativeness and reliability in accounting for variations in population and treatment characteristics [17]. Publication bias was evaluated through the use of funnel plots and Egger’s regression tests. Sensitivity analyses, conducted by iteratively excluding one study at a time, were performed to examine the robustness of the combined findings. All statistical analyses were conducted using R software (v4.2.2) (R Foundation for Statistical Computing, Vienna, Austria), with a significance level set at *p* < .05.

### Kaplan–Meier’s survival analysis on RNA-seq data

To corroborate the results of our meta-analysis, we performed Kaplan–Meier’s survival analyses using RNA-seq data from The Cancer Genome Atlas (TCGA) assessed via the Gene Expression Profiling Interactive Analysis 2 (GEPIA 2) online tool (http://gepia2.cancer-pku.cn/#index) [18]. We focused on four distinct types of urological cancers for this analysis: bladder urothelial carcinoma (BLCA), kidney chromophobe (KICH), kidney renal clear cell carcinoma (KIRC) and kidney renal papillary cell carcinoma (KIRP). The survival analyses were carried out by designating the ‘Group Cutoff’ as ‘Median’ for the *CXCL8* gene (encoding IL-8) expression, thereby stratifying the samples into high and low expression cohorts. Kaplan–Meier’s survival plots were constructed to evaluate and compare OS and disease-free survival (DFS) between these expression groups. The statistical significance of the differences was determined using the log-rank test.

## Results

### Study selection

The initial search yielded 2118 records. After the elimination of duplicates, 1645 studies were retained for preliminary screening. Following a review of the titles and abstracts, 1619 studies were deemed irrelevant and excluded. This left 26 studies for more detailed assessment. Ultimately, a total of 12 studies met all the eligibility criteria and were incorporated into the meta-analysis [19–30]. Fig. S1 illustrates the study selection process.

### Characteristics of the included studies

A total of 2740 patients were enrolled across the studies, with 2740 patients analysed for OS and 1590 patients for PFS. The 12 studies comprised a mix of prospective studies, retrospective studies and *post hoc* analyses. The sample sizes of these studies ranged from 28 to 443 patients. The predominant treatment modalities were targeted therapies and immunotherapies. The cancer types included in the analysis were UC and RCC. The methodological quality of all included studies was rated as ‘high’ based on the modified NOS (further details are provided in Table S2). The characteristics of the included studies are summarized in Table 1.

**Table 1.** Characteristics of studies included in the meta-analysis.

Study	Study design	Patients with IL-8 data (N)	IL-8 cutoff value (pg/mL)	IL-8 detection method	Treatment	Cancer type	Cancer stage	HR for OS (95%CI)	HR for PFS (95%CI)
Necchi et al. [19]	*Post hoc* analysis	41	67.0	ELISA	Pazopanib	UC	Advanced	1.73 (1.09–2.73)	NA
Guida et al. [20]	*Post hoc* analysis	55	50.7	Multiplexed assay	IL-2-based therapy	RCC	Metastatic	1.89 (1.04–3.43)	NA
Harmon et al. [21]	Prospective	31	7.0	ELISA	Sunitinib	RCC	Metastatic	1.37 (0.76–2.48)	1.20 (0.69–2.10)
		29	9.5		IFN-α			1.52 (0.92–2.51)	1.84 (1.06–3.47)
Schalper et al. [22]	*Post hoc* analysis	392	23	Immunoassay	Nivolumab	RCC	Advanced	2.56 (1.89–3.45)	1.36 (1.07–1.72)
Yuen et al. [23]	*Post hoc* analysis	88	15	Immunoassay	1st line atezolizumab	UC	Metastatic	2.71 (1.48–4.97)	NA
		241			2+ line atezolizumab	UC	Metastatic	1.84 (1.27–2.66)	NA
		443			Atezolizumab	UC	Metastatic	1.84 (1.80–2.26)	NA
		88			Atezolizumab	RCC	Metastatic	2.55 (1.18–5.50)	NA
		83			Atezolizumab plus bevacizumab	RCC	Metastatic	1.25 (0.61–2.60)	NA
Bilen et al. [24]	*Post hoc* analysis	53	Median	Luminex bead-based assays	Sunitinib	RCC	Advanced	2.61 (1.03–6.58)	1.54 (0.87–2.72)
Shibata at al. [25]	Respective	57	Median	Bead-based multiplex assay	Pembrolizumab	UC	Advanced	3.40 (1.26–9.20)	2.95 (0.91–9.59)
Powles et al. [26]	*Post hoc* analysis	316	4.60	ELISA or Luminex assay platforms	Cabozantinib	RCC	Advanced	1.77 (1.25–2.50)	1.03 (0.76–1.40)
		304	5.05		Everolimus			1.67 (1.23–2.27)	1.33 (1.01–1.76)
Msaouel et al. [27]	*Post hoc* analysis	37	Median	Immunobead assay	Sunitinib Everolimus	RCC	Advanced	3.55 (1.55–8.14)	3.13 (1.41–6.92)
Tran et al. [28]	*Post hoc* analysis	225	16.42	SearchLight protein array	Pazopanib	RCC	Metastatic	1.50 (1.15–1.96)	1.57 (1.14–2.16)
		118			Placebo			3.34 (1.82–6.13)	2.85 (1.47–5.52)
Maiorano et al. [29]	Prospective	28	3.15	Bead-based cytokine assay	Avelumab	UC	Metastatic	3.13 (1.16–8.41)	3.17 (0.72–13.97)
Jing et al. [30]	Retrospective	111	21.2	Cytometric bead array analysis	Radical cystectomy or transurethral resection	UC	NA	1.68 (1.07–2.64)	NA

IL-8: interleukin-8; HR: hazard ratio; OS: overall survival; PFS: progression-free survival; CI: confidence interval; UC: urothelial carcinoma; RCC: renal cell carcinoma; NA: not available.

### Elevated cIL-8 levels and survival in urological cancer

Harmon et al. [21], Powles et al. [26] and Tran et al. [28] provided HR data on survival outcomes stratified by treatment regimen. In contrast, Yuen et al. [23] supplied HR data that were further delineated by both clinical trial and treatment regimen. Consequently, these studies were processed and amalgamated individually, yielding a total of 19 cohorts contributing HR data for the analysis of OS and 11 cohorts for the analysis of PFS, respectively.

**Figure 1.**
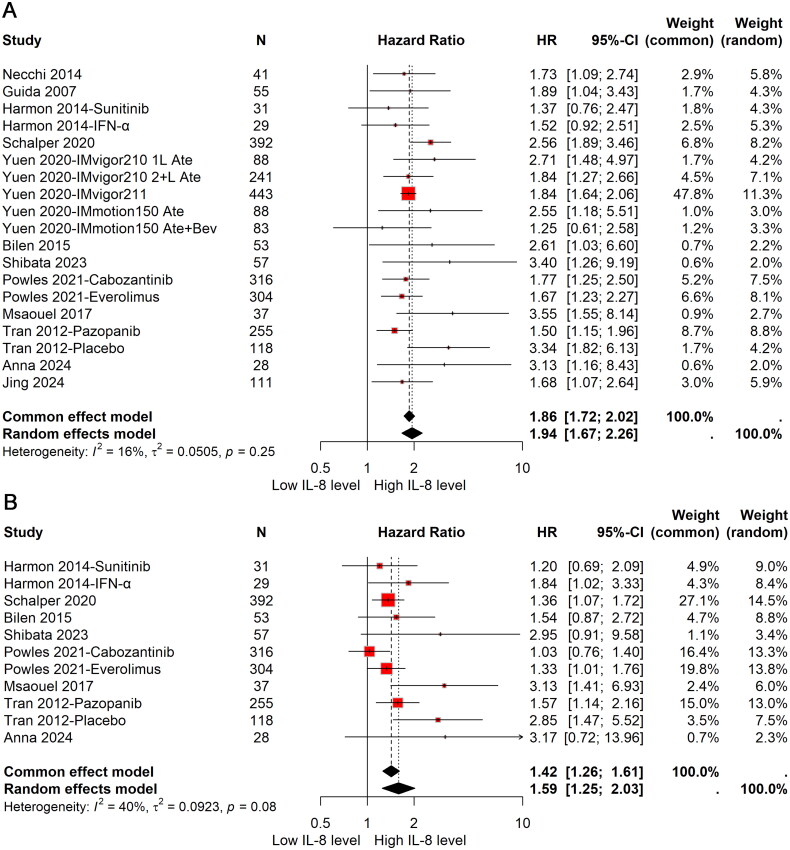
Forest plot of prognostic impact for OS (A) and PFS (B) of cIL-8 levels in urological cancer patients. HR: hazard ratio; OS: overall survival; PFS: progression-free survival.

All studies, encompassing a total of 2740 patients, examined the influence of elevated cIL-8 levels on OS in patients with urological cancers. In light of the negligible heterogeneity observed across the studies (*I*^2^ = 16%), a fixed-effects model was applied to derive the pooled estimate of OS. The meta-analysis indicated that patients with elevated cIL-8 levels had significantly worse OS. Specifically, high cIL-8 levels were associated with an 86% increased risk of mortality (HR: 1.86; 95%CI: 1.72–2.02) (Figure 1(A)), highlighting the detrimental impact of elevated cIL-8 levels on survival in this patient population.

A total of 1590 patients were included in the assessment of the effect of elevated cIL-8 levels on PFS in patients with urological cancers. The meta-analysis indicated that high cIL-8 levels were associated with a 59% increased risk of disease progression compared to patients with low cIL-8 levels (HR: 1.59; 95%CI: 1.25–2.03) (Figure 1(B)). Notably, moderate heterogeneity was present among the studies incorporated into the analysis (*I*^2^ = 40%).

### Subgroup analysis

To systematically evaluate the impact of cIL-8 on the survival outcomes of urological cancers patients, subgroup analyses based on cancer type and treatment regimen were conducted. This stratified method enabled us to explore potential differences in the effects of cIL-8 across various cancer types and treatment modalities, thereby enhancing our understanding of the prognostic significance of cIL-8 in these patient populations.

**Figure 2.**
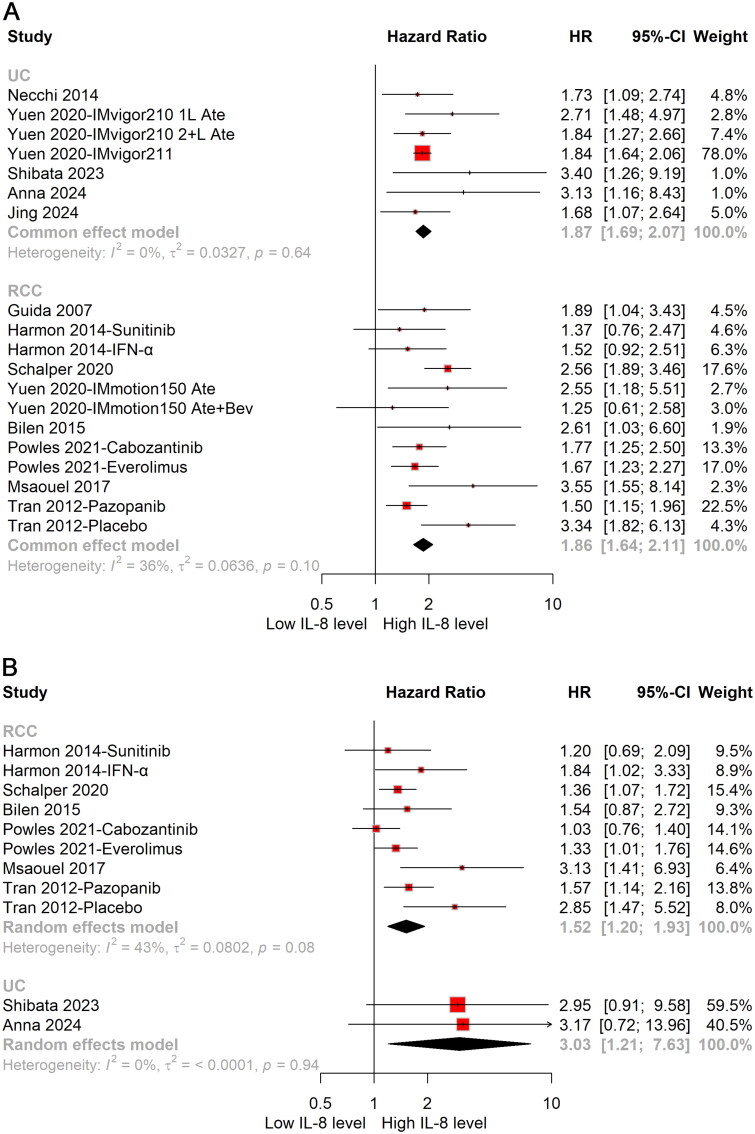
Forest plot of prognostic impact for OS (A) and PFS (B) of cIL-8 levels in urological cancer patients stratified by cancer type. HR: hazard ratio; OS: overall survival; PFS: progression-free survival; UC: urothelial carcinoma; RCC: renal cell carcinoma.

The patient cohort was segmented into two distinct subgroups based on the type of urological cancer. Subgroup analyses revealed that elevated levels of cIL-8 were associated with significantly adverse effects on both OS (HR: 1.87; 95%CI: 1.69–2.07) and PFS (HR: 3.03; 95%CI: 1.21–7.63) in patients with UC (Figure 2). Similarly, high cIL-8 levels were linked to significantly detrimental impacts on both OS (HR: 1.86; 95%CI: 1.64–2.11) and PFS (HR: 1.52; 95%CI: 1.20–1.93) in patients with RCC (Figure 2).

**Figure 3.**
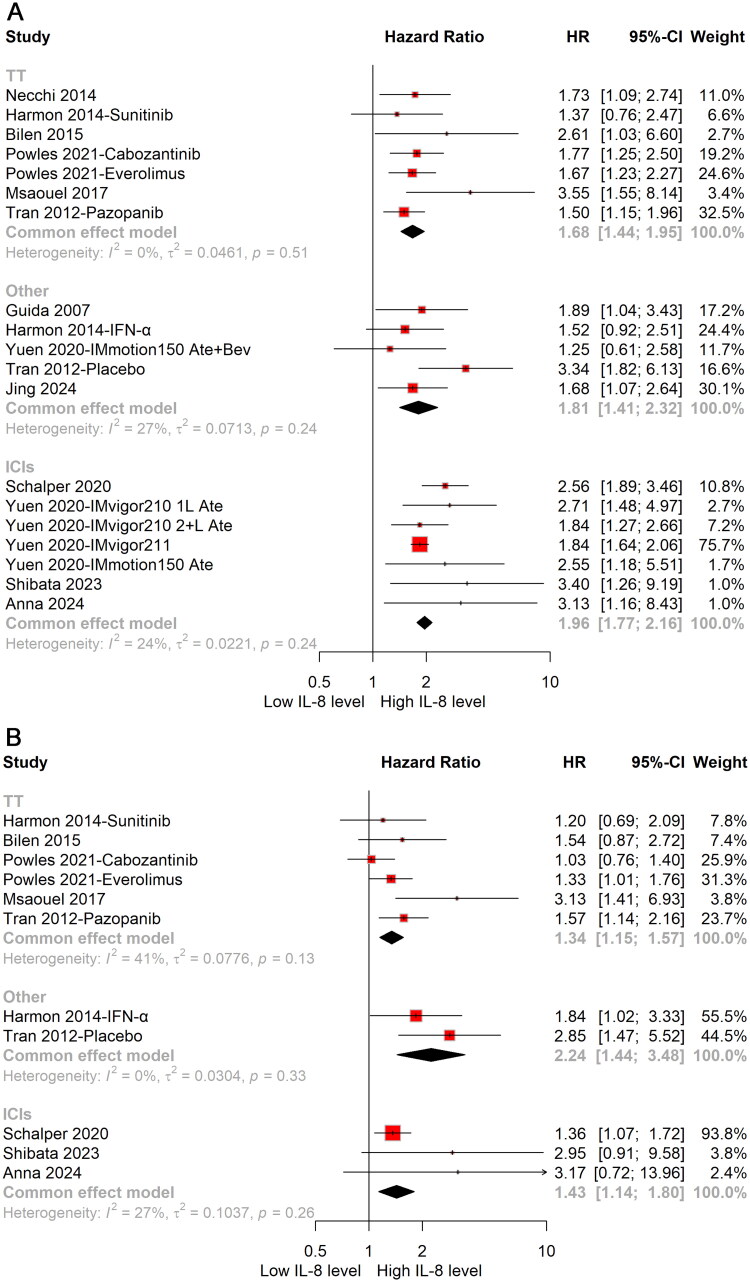
Forest plot of prognostic impact for OS (A) and PFS (B) of cIL-8 levels in urological cancer patients stratified by treatment regimen. HR: hazard ratio; OS: overall survival; PFS: progression-free survival; TT: targeted therapy: ICIs: immune checkpoint inhibitors.

Subgroup analyses, stratified by treatment regimen, have elucidated distinct patterns in the influence of cIL-8 levels on clinical outcomes. High cIL-8 levels were observed to have a detrimental effect on OS in patients receiving targeted therapies (HR: 1.68; 95%CI: 1.44–1.95), immunotherapies (HR: 1.96; 95%CI: 1.77–2.16) and other therapies (HR: 1.81; 95%CI: 1.41–2.32) (Figure 3(A)). Correspondingly, high cIL-8 levels were associated with significantly shorter PFS in patients receiving targeted therapies (HR: 1.34; 95%CI: 1.15–1.75), immunotherapies (HR: 1.43; 95%CI: 1.14–1.80) and other therapies (HR: 2.24; 95%CI: 1.44–3.48) (Figure 3(B)).

### Publication bias and sensitivity analysis

Funnel plot, in conjunction with Egger’s test, indicated the absence of significant publication bias that influenced OS (*p* = .1769) (Fig. S2A). However, significant publication bias was detected in PFS (*p* = .0141) (Fig. S2B). Sensitivity analyses, employing the leave-one-out approach, revealed that none of the individual studies significantly altered the pooled HRs for OS or PFS, indicating the robustness of the results (Fig. S3).

### Bioinformatic validation

**Figure 4.**
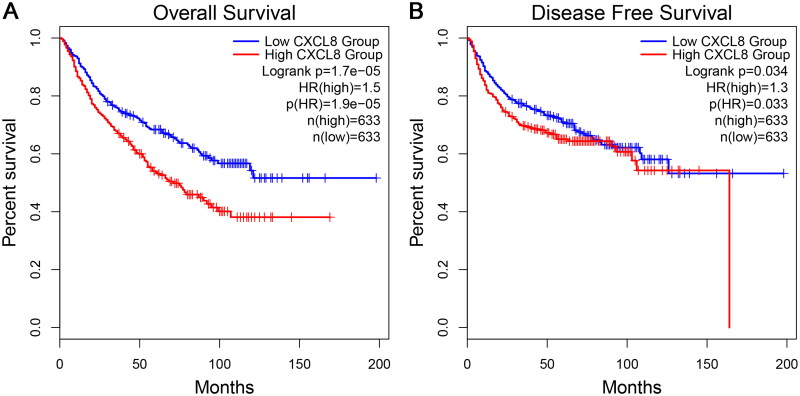
Kaplan–Meier’s curve showing the OS (A) and DFS (B) probabilities of urological cancer patients, using the GEPIA 2 tool, for BLCA, KICH, KIRC and KIRP combined, stratified by high and low expression of *CXCL8*. HR: hazard ratio; OS: overall survival; DFS: disease-free survival; BLCA: bladder cancer; KICH: kidney chromophobe; KIRC: kidney renal clear cell carcinoma; KIRP: kidney renal papillary cell carcinoma.

To corroborate the potential prognostic value of cIL-8, we conducted an analysis of the impact of *CXCL8* gene expression on the OS and DFS of patients with urological cancers, utilizing RNA-seq data from TCGA through GEPIA 2. Following the integration of normalized gene expression data from BLCA (*n* = 402), KICH (*n* = 64), KIRC (*n* = 516) and KIRP (*n* = 282), a total of 1266 patients with urological cancers were analysed. Subsequently, Kaplan–Meier’s curves were plotted to stratify patients based on *CXCL8* expression. Survival analyses revealed that patients with high *CXCL8* expression exhibited significantly reduced OS (HR, 1.50; *p* = 1.7 × 10^−5^) and DFS (HR, 1.30; *p* = .034) compared to those with low expression (Figure 4).

## Discussion

Multiple studies have corroborated the pivotal role of IL-8 as an inflammatory and immunosuppressive factor within the tumour microenvironment [6,7]. IL-8 not only promotes tumour progression and is involved in angiogenesis but also has the capacity to recruit immunosuppressive cells, thereby suppressing the anti-tumour immune response [6,10]. Concurrently, several clinical studies have identified a significant correlation between elevated cIL-8 levels and a poorer prognosis in urological cancer patients [22,26,28]. However, the correlation between elevated cIL-8 levels and prognostic outcomes in urological cancer patients remains equivocal, due to the small sample size of the studies and contradictory results reported in the literature [23]. Consequently, a comprehensive meta-analysis synthesizing data from multiple studies is necessary to clarify this relationship.

Our present meta-analysis, which encompassed 19 cohorts involving a total of 2740 patients, provides insights into the prognostic impact of elevated cIL-8 levels in urological cancer patients. The results of our analysis have revealed promising evidence of cIL-8 as a potential biomarker for predicting treatment response. Moreover, our study is the first to demonstrate that elevated cIL-8 levels are associated with an unfavourable prognostic impact on both OS and PFS in urological cancer patients. Additionally, the robustness of these findings, supported by *in silico* validation, underscores the potential of cIL-8 as a prognostic biomarker in this clinical setting. These results not only validate previous observations suggesting the prognostic relevance of IL-8 in urological cancers but also highlight the urgent need for further exploration of IL-8-targeted therapies. This study significantly contributes to the ongoing debate surrounding the potential biomarker role of cIL-8 in urological cancer patients, particularly in the context of increased drug resistance. Given the increasing significance of identifying predictive biomarkers and therapeutic targets in urological cancer treatment, the findings of our study have profound implications for clinical practice.

To enhance our understanding of the prognostic implications of cIL-8 levels in urological cancer patients, our study utilized a stratified analysis that considered both cancer type and treatment regimen. Subgroup analyses, categorized by cancer type, revealed that elevated cIL-8 levels were associated with adverse effects on both OS and PFS in patients with UC and RCC. This suggests that cIL-8 may serve as a prognostic biomarker across various urological cancer subtypes. When stratified by treatment regimen, high cIL-8 levels were linked to significantly shorter OS and PFS in patients receiving targeted therapies, immunotherapies and other treatment modalities. These findings underscore that high cIL-8 levels correlate with adverse outcomes in urological cancer irrespective of the treatment modalities employed.

IL-8 is increasingly acknowledged for its pivotal role in cancer progression and resistance to a range of therapeutic interventions, including chemotherapy [31], targeted therapies [6] and immune checkpoint inhibitors (ICIs) [10]. Beyond its involvement in treatment resistance, IL-8 also emerges as a promising predictive biomarker. The integration of ICIs into the treatment paradigm for urological cancers has significantly advanced therapeutic strategies [32,33]. However, the identification of patients who will respond to ICIs therapy remains a clinical challenge, leading to a significant proportion of patients failing to benefit from these treatments. This challenge underscores the necessity for improved patient selection for ICIs therapy. Currently, there is a lack of singular ideal biomarker for stratifying patients receiving ICIs therapy, which leads to variability in patient selection. Despite biomarkers such as PD-L1 expression, microsatellite instability and tumour mutation burden (TMB) are frequently employed for predicting therapeutic responses [34], their effectiveness is constrained, prompting the search for alternative biomarkers. Moreover, significant heterogeneity exists in the methodologies employed for biomarker detection. Notably, the assessment of PD-L1 typically employs immunohistochemistry techniques, where the reliability of outcomes is considerably contingent upon the sensitivity and specificity of the staining process [35]. Additionally, TMB analysis might lack universal availability and insurance coverage, which could limit patient access to such critical diagnostic resources [36]. Consequently, it is of utmost importance to identify more potent biomarkers that can accurately predict ICIs responses. Compared to the current biomarkers used for immunotherapy response prediction, cIL-8 levels offer a straightforward quantitative measure. This parameter can be readily quantified from routine blood samples in clinical practice, enhancing its applicability in clinical settings.

Although our meta-analysis did not directly investigate the underlying mechanisms underlying the observed associations, several reasonable explanations can be deduced from the existing literature. First, IL-8 influences tumour biological behaviour through multiple complex pathways [37]. At the cellular level, IL-8 can directly interact with its receptors CXCR1 and CXCR2 on tumour cells [7,37]. This binding triggers a series of intracellular signalling cascades, such as the activation of the PI3K-AKT and MAPK pathways. The activation of the PI3K–AKT pathway is involved in promoting cell survival by suppressing apoptosis and enhancing cellular metabolism. Meanwhile, the activation of the MAPK pathway can result in increased expression of genes related to cell proliferation and motility. Moreover, angiogenesis induced by IL-8 is another critical aspect [37]. It stimulates endothelial cells to proliferate and migrate, giving rise to new blood vessels that supply essential nutrients and oxygen to the tumour, thus facilitating tumour growth. Concurrently, IL-8 recruits immunosuppressive cells into the tumour microenvironment, which can impede effective anti-tumour immune responses [37,38]. IL-8 acts as a potent chemoattractant for various immune cells, including MDSCs, TAMs and neutrophils. MDSCs can dampen the anti-tumour immune response by inhibiting T cell activation and function, while TAMs can adopt a pro-tumorigenic phenotype, promoting tumour cell survival, proliferation and invasion. For instance, TAMs secrete growth factors and proteases that remodel the extracellular matrix, facilitating tumour cell migration. This is especially significant in the context of ICIs, where IL-8-driven myeloid cell infiltration is associated with resistance. Understanding these detailed mechanisms is of paramount importance as it can offer valuable insights for the development of targeted therapies against IL-8 or its associated signalling pathways. By precisely targeting these processes, we may potentially disrupt the tumour-promoting effects of IL-8 and improve the prognosis of urological cancer patients. Future studies should concentrate on further elucidating these mechanisms in greater depth and exploring potential therapeutic targets based on this knowledge.

Despite the strength of our findings, several limitations should be acknowledged. First, the majority of the included studies were *post hoc* analyses, which may introduce selection biases. To overcome these limitations, future research endeavours should be meticulously executed through the utilization of well-structured prospective cohort studies or randomized controlled trials. These types of investigations would not only furnish more robust evidence regarding the causal relationship between cIL-8 levels and outcomes in urological cancers but also play a crucial role in validating the results of our current study. Moreover, the variation in cIL-8 cut-off values among studies indeed poses a challenge to the direct comparison of results. Different cut-off values might lead to heterogeneity in patient classification, potentially influencing the observed associations between cIL-8 levels and survival outcomes. For instance, a higher cut-off value could result in a smaller proportion of patients being categorized as having elevated cIL-8 levels, thereby altering the magnitude of the effect on prognosis. Future research should focus on establishing standardized measurement techniques and consensus cut-off values. This could involve large-scale multicentre studies with a unified assay method to determine the optimal threshold that maximizes the prognostic value of cIL-8. By doing so, we can enhance the comparability of results across different studies and improve the reliability of cIL-8 as a biomarker. Lastly, while there was no significant publication bias detected for OS, a significant bias was identified for PFS. This bias is likely due to the preference for publishing studies with positive or significant results, often resulting in the disregard or delay of those with negative or non-significant ones. Smaller studies showing negative or less significant associations between cIL-8 and PFS are less likely to be published, which may lead to an overestimation of the true effect. Hence, when interpreting PFS results, caution is necessary as the observed effect might be inflated. To tackle this issue in future research, it is essential to encourage prospective registration of all cIL-8 and urological cancer studies and ensure the publication of all findings regardless of the outcome. This will contribute to a more comprehensive and unbiased understanding of the relationship between cIL-8 and PFS.

## Conclusions

This meta-analysis offers valuable insights into the potential prognostic significance of cIL-8 in urological cancers. The data suggest that elevated levels of cIL-8 may serve as a biomarker for poorer OS and PFS in patients with these malignancies. While the findings highlight the potential of cIL-8 as a therapeutic target, it is important to acknowledge the limitations of the current study. The reliance on retrospective data, variability in IL-8 measurement methods, and the possibility of publication bias for PFS outcomes introduce caveats to the generalizability and robustness of our findings. Consequently, the clinical utility of cIL-8 as a biomarker remains to be fully validated. Future research, including prospective cohort studies and randomized controlled trials, is essential to establish the predictive value of cIL-8 and to develop standardized protocols for its use in clinical practice.

## Supplementary Material

Supplemental Material

## Data Availability

The data resulting from this study, including all generated or analysed datasets, are contained within the published article and its supplements. For any additional data-related inquiries, the corresponding author is available to address reasonable requests upon contact.
