# Supplementary material for: Elevated circulating IL-8 correlates with poor prognosis in urological cancers: a meta-analysis and bioinformatic validation
Source: Ann Med. 2025 Apr 3;57(1):2486592. doi: 10.1080/07853890.2025.2486592 (PMC11980208; doi:10.1080/07853890.2025.2486592)
Supplement: Supplemental Material [file IANN_A_2486592_SM8614.zip › Supplementary Material/Supplementary Material (44).docx]

**Supplementary Material**

**Elevated circulating IL-8 correlates with poor prognosis in urological cancers: A meta-analysis and bioinformatic validation**

**Table S1.** Details of searching strategy.

**Table S2.** Quality assessment of included studies through the modified Newcastle-Ottawa Quality Assessment Scale (NOS).

**Fig S1.** PRISMA flow diagram of study selection.

**Fig S2.** Publication bias assessment for included studies on overall survival and progression-free survival using funnel plots with Egger's regression tests.

**Fig S3.** Sensitivity analyses for included studies on overall survival and progression-free survival examined by leaving-one-out approach.

**Table S1.** Details of searching strategy.

| **Database** | **Search strategy** |
| --- | --- |
| ***PubMed*** | ((((("Interleukin-8"[Mesh]) OR ("Interleukin 8")) OR (IL-8)) OR ("IL 8")) OR (CXCL8)) AND ((((("Carcinoma, Transitional Cell"[Mesh]) OR ("Transitional Cell Carcinoma")) OR ("Urothelial Carcinoma")) OR ("Bladder Cancer")) OR ((((("Carcinoma, Renal Cell"[Mesh]) OR ("Renal Cell Carcinoma")) OR ("Renal Adenocarcinoma")) OR ("Kidney Cancer")) OR ("Clear Cell Carcinoma"))) |
| ***Scopus*** | TITLE-ABS-KEY((((("Interleukin-8") OR ("Interleukin 8")) OR ("IL-8")) OR ("IL 8")) OR ("CXCL8")) AND TITLE-ABS-KEY((((("Transitional Cell Carcinoma")) OR ("Urothelial Carcinoma")) OR ("Bladder Cancer")) OR ((((("Renal Cell Carcinoma")) OR ("Renal Adenocarcinoma")) OR ("Kidney Cancer")) OR ("Clear Cell Carcinoma"))) |
| ***Embase*** | ('interleukin-8'/exp OR 'interleukin 8' OR 'il-8' OR 'il 8' OR 'cxcl8') AND ('urothelial carcinoma'/exp OR 'renal cell carcinoma'/exp OR 'bladder cancer' OR 'renal adenocarcinoma' OR 'kidney cancer' OR 'clear cell carcinoma') |

**Table S2.** Quality assessment of included studies through the modified Newcastle-Ottawa Quality Assessment Scale (NOS).

| **Items Studies** | Necchi 2014 | Guida 2007 | Harmon 2014 | Schalper 2020 | Yuen 2020 | Bilen 2015 | Shibata 2023 | Powles 2021 | Msaouel 2017 | Tran 2012 | Anna 2024 | Jing 2024 |
| --- | --- | --- | --- | --- | --- | --- | --- | --- | --- | --- | --- | --- |
| **Selection** |  | | | | | | | | | |  |  |
| Representativeness of the exposed cohort | 1 | 1 | 1 | 1 | 1 | 1 | 1 | 1 | 1 | 1 | 1 | 1 |
| Selection of the non-exposed cohort | 1 | 1 | 1 | 1 | 1 | 1 | 1 | 1 | 1 | 1 | 1 | 1 |
| Ascertainment of exposure | 1 | 1 | 1 | 1 | 1 | 1 | 1 | 1 | 1 | 1 | 1 | 1 |
| Demonstration that outcome of interest was not present at the start of study | 1 | 1 | 1 | 1 | 1 | 1 | 1 | 1 | 1 | 1 | 1 | 1 |
| **Compatibility** |  | | | | | | | | | | | |
| Comparability of cohorts on the basis of the design or analysis | 1 | 1 | 1 | 1 | 1 | 1 | 1 | 1 | 1 | 1 | 1 | 1 |
| **Assessment** |  | | | | | | | | | | | |
| Assessment of outcome | 1 | 1 | 1 | 1 | 1 | 1 | 1 | 1 | 1 | 1 | 1 | 1 |
| Was follow-up long enough for outcomes to occur | 1 | 1 | 1 | 1 | 1 | 1 | - | 1 | 1 | 1 | 1 | - |
| Adequacy of follow up of cohorts | 1 | 1 | 1 | 1 | 1 | 1 | 1 | 1 | 1 | 1 | 1 | 1 |
| **Total score** | 8 | 8 | 8 | 8 | 8 | 8 | 7 | 8 | 8 | 8 | 8 | 7 |


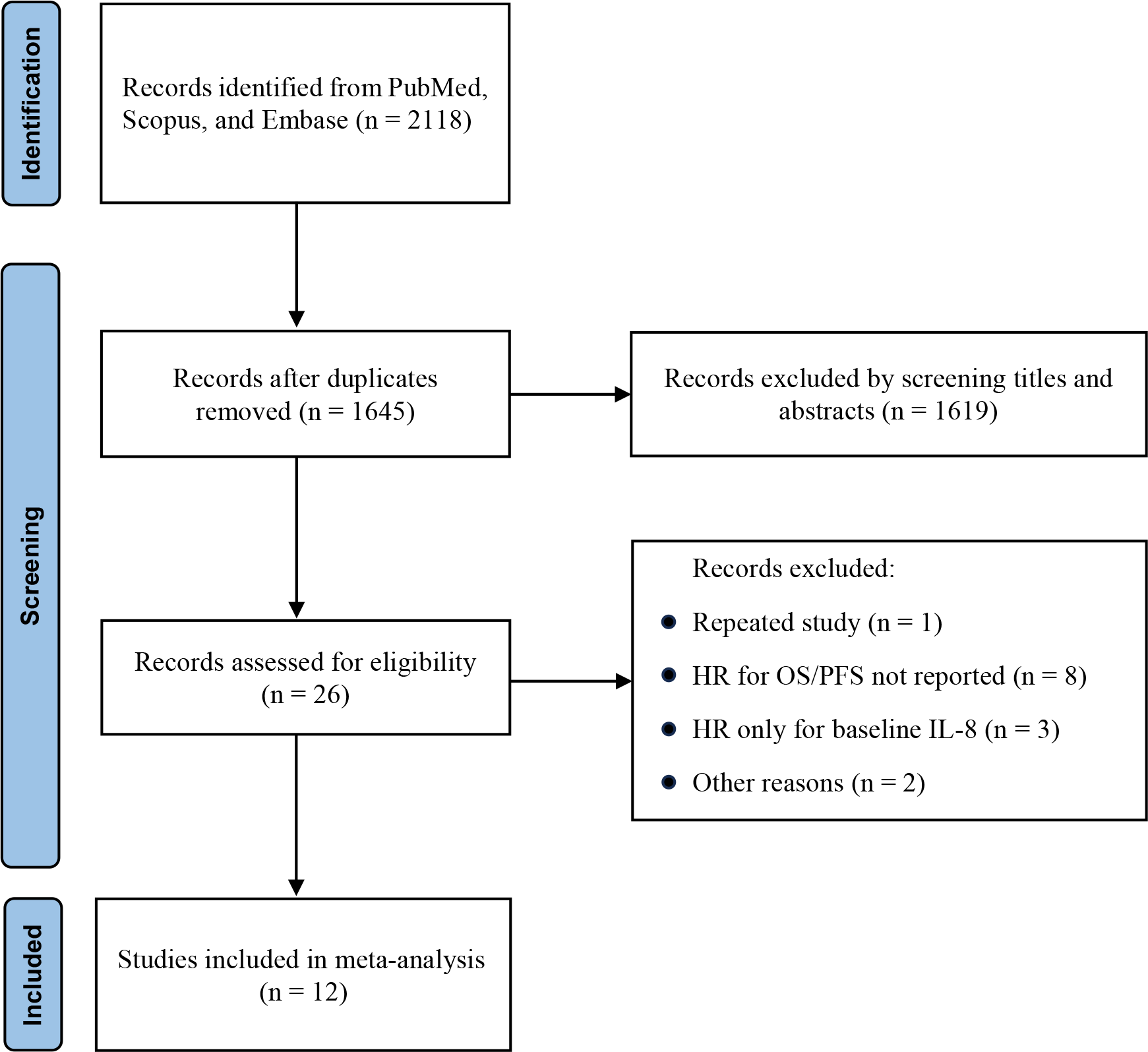


**Fig S1.** PRISMA flow diagram of study selection. Abbreviations: HR, hazard ratio; OS, overall survival; PFS, progression-free survival.

**
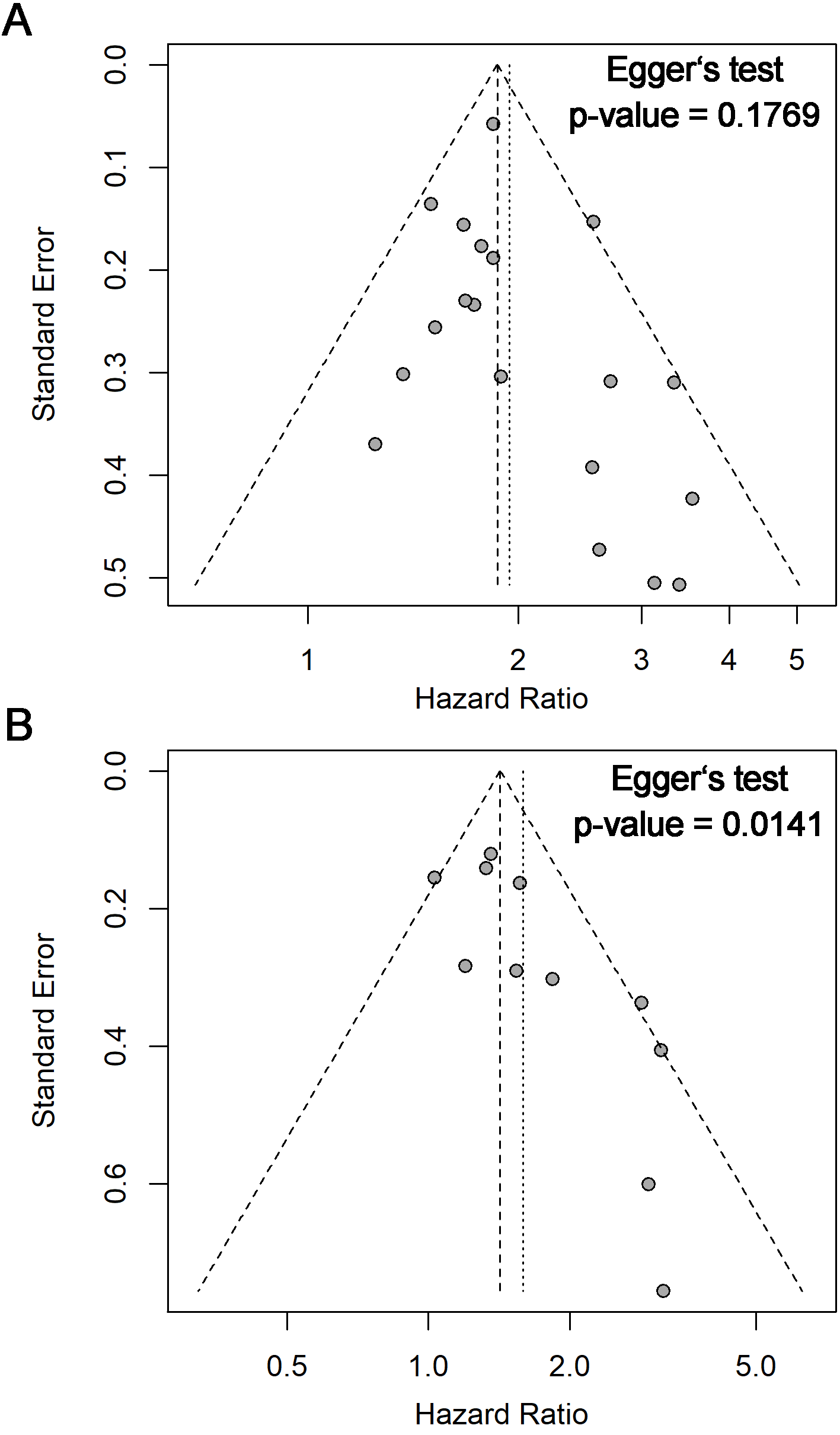
Fig S2**. Publication bias assessment for included studies on overall survival (**A**) and progression-free survival (**B**) using funnel plots with Egger's regression tests.

**
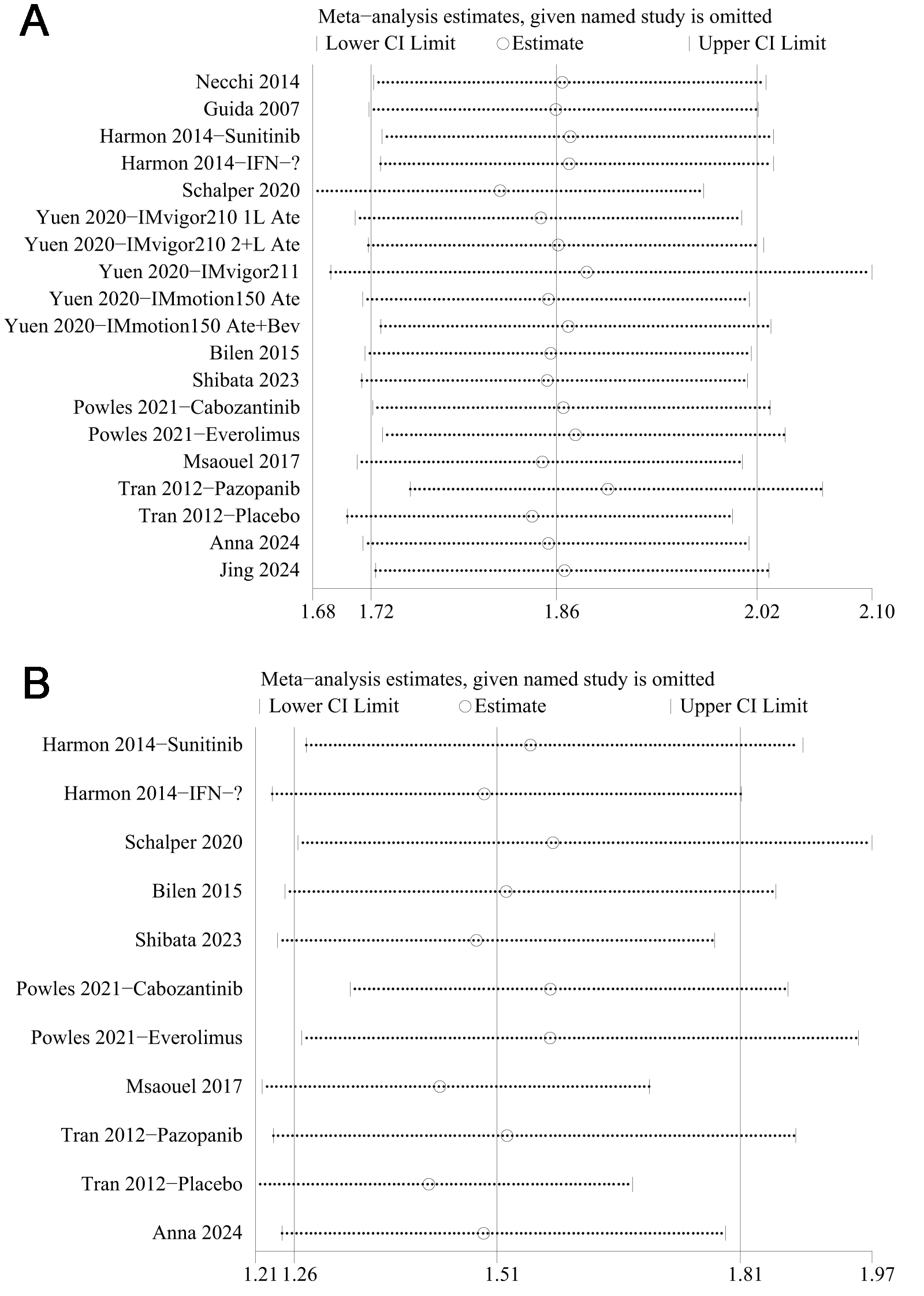
Fig S3**. Sensitivity analyses for included studies on overall survival (**A**) and progression-free survival (**B**) examined by leaving-one-out approach. Abbreviations: CI, confidence interval.
